# Dynamic Internal Jugular Vein Compression by Hypertrophic Hyoid Bone: Management and Outcomes

**DOI:** 10.7759/cureus.7445

**Published:** 2020-03-28

**Authors:** Soliman Oushy, John T Wald, Jeffrey Janus, Jimmy R Fulgham, Giuseppe Lanzino

**Affiliations:** 1 Neurological Surgery, Mayo Clinic, Rochester, USA; 2 Radiology, Mayo Clinic, Rochester, USA; 3 Otorhinolaryngology, Mayo Clinic, Jacksonville, USA; 4 Neurology, Mayo Clinic, Rochester, USA

**Keywords:** internal jugular vein, compression, hyoid bone, dynamic, atypical facial pain

## Abstract

Extracranial osseous compression of the internal jugular vein (IJV) is exceedingly rare. The clinical manifestations of IJV obstruction are very heterogeneous and subtle, and arriving at a diagnosis can be challenging. We describe a case of dynamic IJV compression in a 40-year-old male with progressive, positional, ill-defined right periorbital and neck pain associated with photosensitivity. Imaging showed a hypertrophic right hyoid bone; computed tomography venogram (CTV) with challenging maneuvers demonstrated dynamic compression of the ipsilateral IJV by a hypertrophied hyoid bone and thyroid cartilage. The patient underwent decompression of the right jugular vein which resulted in the resolution of his symptoms. The clinical manifestations of extracranial IJV impingement are variable and diagnostically challenging. Disturbances in extracranial IJV outflow is a diagnosis of exclusion and could be responsible for atypical facial pain in a select group of patients. This entity should be considered in the differential of atypical facial, especially when symptoms tend to be positional.

## Introduction

Disturbances in extracranial internal jugular vein (IJV) outflow are increasingly being recognized as a cause of various clinical conditions such as idiopathic intracranial hypertension (IIH). While the clinical sequelae of disrupted arterial flow present with a predictable pattern, symptoms related to disruption of venous outflow are highly variable and difficult to diagnose early [1]. We present a case of dynamic unilateral IJV compression presenting with atypical facial and neck pain. We discuss the management and outcomes in this patient population.

## Case presentation

**Figure 1:**
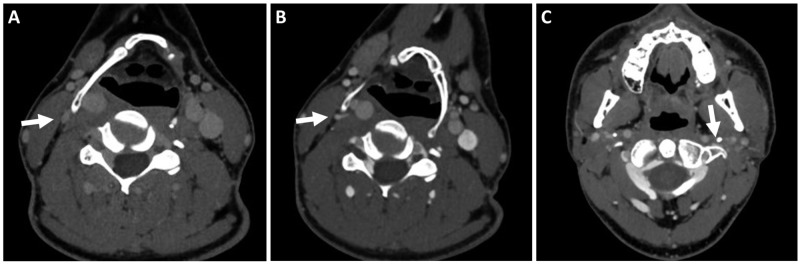
Preoperative axial CTV. (A, B) preoperative axial CTV images showing compression of the right IJV (white arrows) by the hyoid bone in (A) neutral head position and worsening compression on (B) left head turn. (C) Axial CTV demonstrating compression (white arrow) of the contralateral distal IJV between the styloid process and transverse process of first cervical vertebrae. CTV, computed tomography venogram; IJV, internal jugular vein

**Figure 2:**
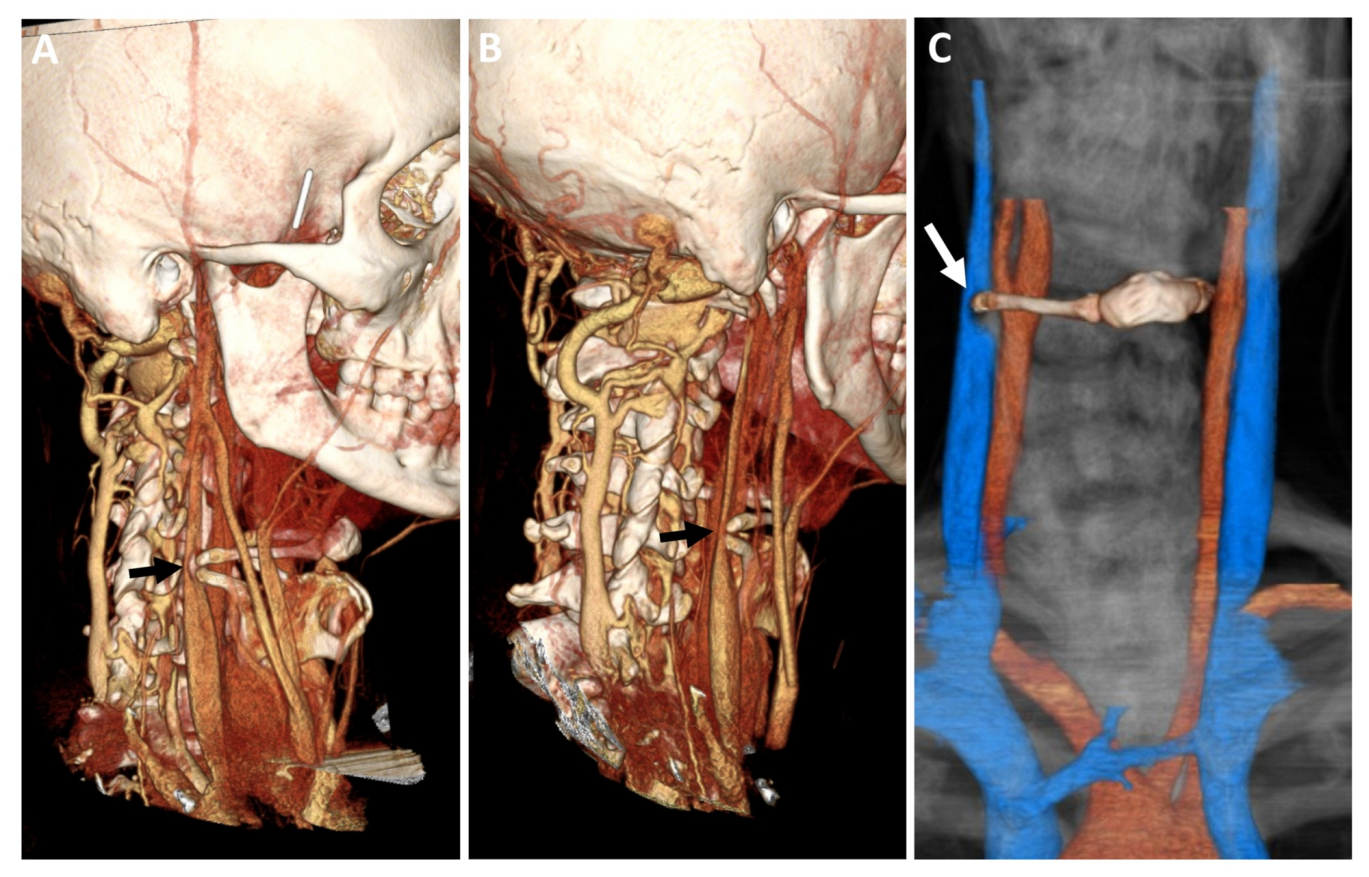
Three-dimensional reconstruction of the preoperative vessel imaging. (A, B, C) Three-dimensional reconstruction of the preoperative CTV clearly demonstrating extrinsic compression of the IJV by a hypertrophied hyoid bone and superior thyroid cartilage (black arrow in A, B; white arrow in C). CTV, computed tomography venogram; IJV, internal jugular vein

A 40-year-old male presents with complaints of ill-defined right periorbital pain associated with intermittent photosensitivity. His symptoms progressed to involve discomfort in the right upper neck. He had no focal neurological deficits on detailed cranial nerve testing. A formal ophthalmology evaluation was negative for papilledema and visual disturbances. He noted symptomatic relief from applying pressure on the lateral aspect of his right neck at the level of the hyoid bone, and he was noted to “readjust” his neck constantly for symptomatic relief during the clinical visit. The symptoms were refractory to medical therapy. Staring at a computer screen had become increasingly uncomfortable, interfering with his daily occupation. He recalled falling face down while playing soccer several weeks prior to symptom onset. MRI of the brain was normal. MRI of the cervical spine revealed age-appropriate mild degenerative changes and diminutive venous caliber in the neck. A computed tomography venogram (CTV) demonstrated enlarged paravertebral and suboccipital plexus, right worse than left, and dynamic compression of the ipsilateral extracranial IJV by a hypertrophic hyoid bone and thyroid cartilage, worsened with leftward head turn in the presence of contralateral IJV compression in the upper neck (Figure 1). The focus of IJV compression, as well as hypertrophy of the paravertebral venous plexus is well illustrated on 3D reconstructions (Figure 2).

**Figure 3:**
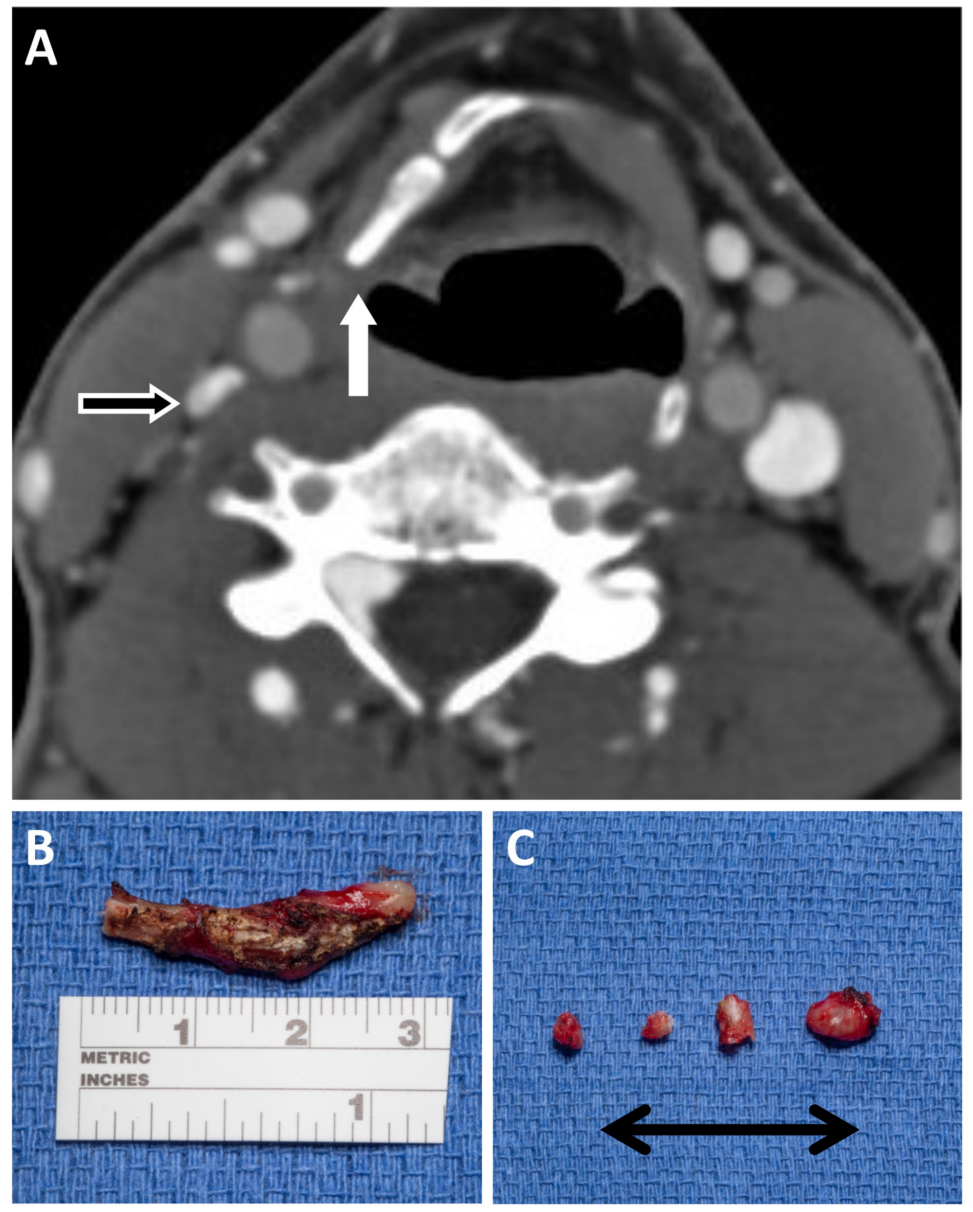
Postoperative venogram and intraoperative pathology. Postoperative axial CTV image showing resection of the hypertrophied segment of the hyoid bone (white arrow) and decompression of the right IJV (black and white arrow). (B, C) Intraoperative images showing resected sections of the (B) hyoid bone measuring nearly 3 cm and (C) fragments of the resected thyroid cartilage. CTV, computed tomography venogram; IJV, internal jugular vein

**Figure 4:**
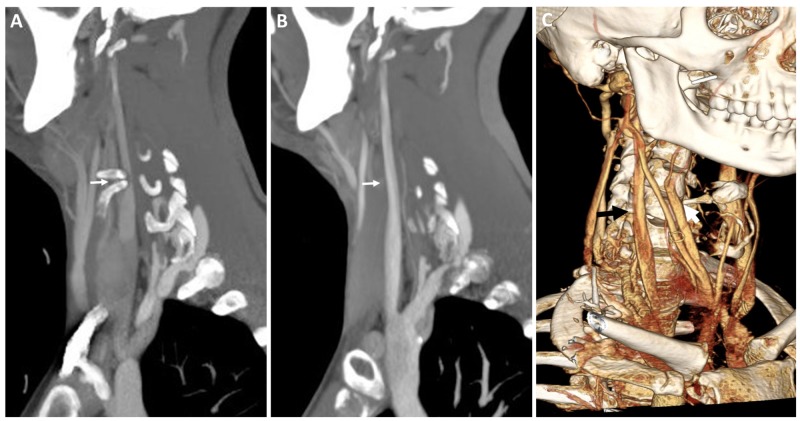
Three-dimensional reconstruction of postoperative vessel imaging. (A) Preoperative and (B) postoperative sagittal CTV images showing resolution of osseous compression (white arrows) by the hyoid bone. (C) Three-dimensional reconstruction of the postoperative CTV showing successful restoration of the IJV caliber (black arrow) and extent of hyoid resection (white arrow). CTV, computed tomography venogram; IJV, internal jugular vein

After extensive counseling and exclusion of alternative possible explanations of his disabling symptoms, it was decided to proceed with decompression of the right IJV via an anterior approach. After induction of anesthesia, a transverse right-sided incision was made following a natural skin crease to expose the posterior half of the greater horn of the hyoid, which was then bisected, and the posterior free portion was disconnected from the thyrohyoid ligament and excised. The superior horn of the thyroid cartilage was also resected to adequately decompress the internal jugular vein (Figure 3). There were no immediate postoperative complications and the patient was discharged home the same day. At a three-month follow-up visit, he reported improvement in his symptoms and return to work full-time. A repeat CTV confirmed the patency of the IJV, both in neutral position and with head turning (Figure 4). 

## Discussion

The clinical manifestations of IJV compression are insidious and frequently heterogeneous. The current understanding and body of literature on this topic is limited to isolated case reports and small clinical series [1-6]. We describe a patient with new onset of atypical, ill-defined facial pain, resolving with decompression of the extracranial IJV by a hypertrophic hyoid bone.

Intracranial venous outflow obstruction has been linked to idiopathic intracranial hypertension (IIH) [7]. Extracranial IJV compression has been linked to several disease entities including multiple sclerosis , migraine headaches, and transient global amnesia [8-10]. However, the exact etiology and significance of venous compression outside the realm of IIH remains controversial. Most cases of extracranial venous compression are asymptomatic due to extensive collateral venous channels. Furthermore, incidental extrinsic compression of the superior extracranial IJV is surprisingly common [11]. Jayaraman et al. reviewed neck CT angiograms in 108 patients and found that extrinsic compression of the IJV (foramen magnum to C3) in unselected patients is unlikely to be pathologic in nature, although there is no mention of a compressive hyoid bone in their series [11]. Locoregional anatomical factors (for example occlusion/hypoplasia of the contralateral IJV) or physiological conditions leading to positional reduction in venous outflow are necessary to trigger symptoms in a few individuals. Our patient exhibited hypertrophy of bilateral paravertebral plexuses presumably in response to osseous compression of bilateral IJV at the level of C1. Further compression of the proximal right IJV by a hypertrophic hyoid, as well as the reactive changes and deformation stemming from the blunt trauma he had suffered, could have resulted in a rather acute change in anatomic relationships triggering symptom insurgence.

Anatomic impingement of the upper IJV, when present, is most commonly observed between the digastric muscle and an adjacent cervical transverse process. Reports of symptomatic osseous impingement of the extracranial IJV in the literature are mostly composed of styloidogenic jugular venous compression syndrome (SJVCS) [2-6]. Osseous compression by a hypertrophic hyoid bone, to our knowledge, has not been observed before in the literature. Most patients with SJVCS presented with complaints of headaches followed by blurry vision, which is hypothesized to be due to elevated intracranial venous pressure as measured by venous manometry, especially in cases with bilateral IJV impingement. Positional headaches related to neck flexion has been shown to be a unique feature of SJVCS, and likely other osteogenic compression as seen in our case [4]. We hypothesize that applying pressure to the anterio-lateral aspect of the neck, our patient reduced the degree of osteogenic compression from the hyoid, therefore improving IJV outflow and his symptoms. Our case stresses the critical necessity of detailed history in identifying venous compression as a possible cause of otherwise common symptoms, often ascribed to idiopathic etiologies.

Management and outcomes

Successful treatment of IJV osseous impingement is dependent on accurate diagnosis and adequate treatment [1]. Head and neck CTV with 3D reconstruction can effectively demonstrate the extrinsic osseous foci of IJV stenosis. CTV with and without head turning can unmask dynamic compression of the CTV. It is important to note that ultrasound is more sensitive in screening and MRI venograms are superior in detecting IJV compression, however, both lack the ability to show osseous compression [12-13]. Endovascular venography with manometry can be used to confirm the diagnosis by measuring pressure gradient across the stenosis and global intracranial venous pressure, which are often elevated in cases of osseous stenosis [4]. Endovascular venography and manometry were deemed unnecessary in our case given the clear dynamic presentation seen with head turning and the exquisite anatomical detail obtained with both static and dynamic CT-based imaging.

Endovascular IJV stenting is not as effective as surgery in cases of osseous stenosis. In fact, stenting alone was shown to be ineffective and may exacerbate the degree of outflow obstruction [6]. Surgical decompression and resection of osseous overgrowth has been applied in cases of SJVCS with excellent outcomes. The largest series by Zhao et al. observed symptomatic improvement in nine out of ten patients with SJVCS treated with surgical styloidectomy and C1 tuberculectomy [4].

## Conclusions

The manifestations of head and neck venous outflow compromise are not well characterized compared to pathological arterial compromise, which result in predictable and reproducible clinical manifestations. Our case highlights venous compromise as a potential cause of atypical facial pain. Dynamic CT venogram of the head and neck with 3D reconstruction can reveal the focus of impingement. Careful history taking, with attention to aggravating or alleviating maneuvers, as well as an assessment of venous pathways should be considered in patients with atypical facial and neck pain suspected to have a possible venous compression.
